# Hepatitis B Virus (HBV) Genotypes in an Ecuadorian Population: A Preliminary Study

**DOI:** 10.1155/2024/8823341

**Published:** 2024-08-23

**Authors:** Miguel Moncayo, Enrique Teran, Bernardo Gutierrez, Jorge Reyes, Johanna Cortez, Rodrigo Tobar, Gabriela Yerovi, Marcia Robalino, Ana Aguilar, Daniel Garzon-Chavez

**Affiliations:** ^1^ Universidad San Francisco de Quito USFQ, Quito, Ecuador; ^2^ Laboratorio Clínico Dr. Alberto Moncayo Calero, Quito, Ecuador; ^3^ University of Oxford, Oxford, UK; ^4^ Universidad Central del Ecuador, Quito, Ecuador; ^5^ Pontificia Universidad Catolica del Ecuador, Quito, Ecuador; ^6^ Ministerio de Salud Pública del Ecuador, Quito, Ecuador

## Abstract

Chronic hepatitis B virus (HBV) infection affects 257–291 million people worldwide. The World Health Organization reported 890,000 HBV-related deaths in 2019, higher than reported previously. There are 10 HBV genotypes (A–J) subdivided into several subgenotypes that differ considerably by geography. Various virologic factors, including genotype and subgenotype, impact the odds of acquiring a chronic HBV infection, the type of treatment prescribed, and the risk of developing hepatocarcinoma. Information on the HBV genotypes and subgenotypes that circulate in Ecuador remains low. To address this gap, the current study took a preliminary look at HBV-infected human samples from this region to identify the most common genotypes and subgenotypes. Samples from 44 patients in the Andean, Coastal, and Amazon regions of Ecuador were amplified and two major genotypes were identified, genotype F (42/44; 95.5%) and genotype E (2 patients; 4.5%). The genotype F subgenotypes were F3 (35/42; 83.33%), F4 (6/42; 14.28%), and F1b (1/42, 2.39%). This is the first epidemiological study to assess the distribution of HBV genotypes in Ecuador. The findings can inform antiviral drug effectivity studies specific to HBV genotypes prevalent in South America.

## 1. Introduction

Hepatitis B virus (HBV) causes an acute infection in humans that can develop into a chronic disease that eventually leads to liver cirrhosis and hepatocellular carcinoma (HCC), depending on the patient's age at the time of infection [1]. In 2019, the World Health Organization (WHO) reported a global HBV prevalence of 3.5% and approximately 890,000 deaths in the general population, higher than reported in prior years [2]. HBV is an enveloped virus with a partially double-stranded DNA genome composed of 3,200 base pairs. The genome includes a coding negative sense strand containing four overlapping open reading frames (ORFs): P, X, preS/S, and preC/C. The preS/S ORF (nt 2848–155) encodes three enveloped glycoproteins: small (S), middle (M), and large (L) surface antigen (HBsAg), which are critical for the diagnosis and identification of specific HBV genotypes [1, 3].

Humans can be infected with 10 distinct HBV genotypes, A–J [4], with significant variation in their geographic distribution [5]. Genotypes C, D, and F are associated with a higher risk of developing cirrhosis and hepatocellular carcinoma than genotypes A and B [5]. In addition, mutations in some genotypes correlate with evasion from immunoglobulin or vaccine-based immunity [5]. While genotype D predominates in Eastern Europe, North Africa, and in West, North, South, and Central Asia, genotypes A and D are the most prevalent in Northwestern Europe. Genotype C is most common in East Asia and Oceania, followed by genotype B, and genotypes E and A are predominant in East, Middle, and South Africa (sub-Saharan Africa) [6]. Genotypes A, B, C, and D are most common in North America [6]. Although genotype F is the most prevalent in Latin America, there is intercountry variation, with genotypes A and D being the most prevalent in Brazil and genotype H having the highest prevalence in Mexico [6]. The predominant HBV genotypes in Ecuador, however, remain unknown. The current study provides a preliminary assessment of the HBV genotypes and subgenotypes circulating in this country.

## 2. Materials and Methods

This was a cross-sectional and descriptive study utilizing an official Ministry of Public Health of Ecuador (MSP) database of all individuals with a confirmed diagnosis of HBV infection. All subjects who (1) had a previously confirmed diagnosis of HBV and were registered in the MSP database, (2) were ≥18 years of age, and (3) provided written informed consent to participate in the study were included.

Since official epidemiologic data on HBV are unavailable, a sample size calculation was performed based on a 95% confidence level and a margin of error of 5%, considering a population prevalence of 2.9% [7]. The total population of Ecuador is 18 million; thus, a sample of 44 was required. This sample size calculation was conducted using the stat calc module of EpiInfo version 7.2 [8, 9].

A total of 451 eligible HBV patients were identified by the MSP from 2017 to 2019. These individuals were invited to join the study until the predetermined sample size was reached. Inaccurate contact information, nonresponsiveness, migration due to the COVID-19 pandemic, refusal to participate, or early demise prevented the enrolment of some cases.

A 10-cc venous blood sample was obtained from each subject at their personal residence and stored in a tube without additives. The samples were promptly centrifuged, and 500 *μ*l of plasma was transferred to Eppendorf tubes. The samples were transported in liquid nitrogen to the central laboratory at Universidad San Francisco de Quito and stored at −80°C until further analysis.

### 2.1. HBV DNA Extraction and preS Gene Amplification

DNA was extracted from the samples using the MagMAX™ Viral/Pathogen Nucleic Acid Isolation Kit (ThermoFisher Scientific Inc).

The HBV preS gene from each sample was amplified using the Platinium™ II Hot-Start Green PCR Master Mix (ThermoFisher Scientific Inc), and the primer sets, PF 5′ TTG GAC TCA CAA GGT GGG AA 3′, PR 5′ GTC CAC CAC GAG TCT AGA CTCT 3′, NF 5′ TCA TTT TGT GGG TCA CCA TAT 3′, and NR 5′ CTG TAA CAC GAG CAG GGG T 3′, to obtain a fragment of 578 bp (4). The amplification mixture included 2 *μ*l of HBV DNA, 0.4 *μ*l of each primer (PF, PR), 4 *μ*l of enhancer, and 3.2 *μ*l of water. The PCR conditions included 35 cycles at 94°C for 5 min, 94°C for 30 sec, 51°C for 30 sec, and 72°C for 45 sec, followed by 72°C for 10 min. An additional PCR cycle was used for any samples that failed to amplify. The resultant amplicons were amplified using nested PCR with the second set of primers (NF-NR). The conditions included 35 cycles at 94°C for 5 min, 94°C for 30 sec, 54°C for 30 sec, and 72°C for 45 sec, followed by 72°C for 10 min. The amplicons were then quantified using an EPOCH Microplate Spectrophotometer Reader and subjected to Sanger sequencing (Macrogen Inc., Seoul, Republic of Korea). The obtained nucleotide sequences were deposited in GenBank under the access numbers: OQ920006–OQ9220076. The correlation between the samples and their accession numbers is shown in Supplementary Table 1.

### 2.2. Phylogenetic Analysis

To identify the specific genotypes, the obtained sequences were compared to homologous sequences in GenBank to identify regions with local similarity using BLAST search at the National Center for Biotechnology Information (NCBI). To assess the genetic diversity of HBV in Ecuador compared to other locations, sequences from GenBank at NCBI were retrieved using the keywords (“country name”) AND (“hepatitis B” or “HBV”) AND (“complete genome”). All South American countries from the continental territory were included in the search. Only sequences with complete genomes (corresponding to a length of 3.2 kb) were included, resulting in the recovery of 481 HBV complete genomes. Multiple alignments were performed using a global alignment with free end gaps in Geneious Prime 2022.0.1 and trimmed to 612 nt to include the preS gene fragment. The final multiple-sequence alignment (MSA) contained 481 HBV preS sequences. Detailed phylogenetic analyses of key genotypes were performed on separate MSAs. For genotype E, additional sequences from African countries were retrieved from GenBank and added to the tree analysis. Maximum likelihood (ML) phylogenetic trees were estimated with IQ-Tree 2.2.0 [10] using a General Time-Reversible (GTR) substitution model with rate heterogeneity across sites modeled as a Gamma distribution with four categories. An HBV sequence from genotype J (AB486012), which corresponds to a divergent genotype closely related to isolates from nonhuman primates, was included as an outgroup [11]. Phylogenetic uncertainty was estimated using an ultrafast bootstrap approximation with 1000 replicates [12] and the Shimodaira–Hasegawa approximate likelihood ratio test [13].

## 3. Results

The study participants had a mean age of 32 ± 16.2 years, and the majority were female (*n* = 26; 59.1%). The ethnicity of the subjects was predominantly mestizo (63.6%; Table 1), and the largest proportion was from Quito (36.4% of the total (Andean) region)), followed by Portoviejo and El Coca (each 11.3%), located in the Coastal and Amazon regions, respectively (Table 1)

**Figure 1:**
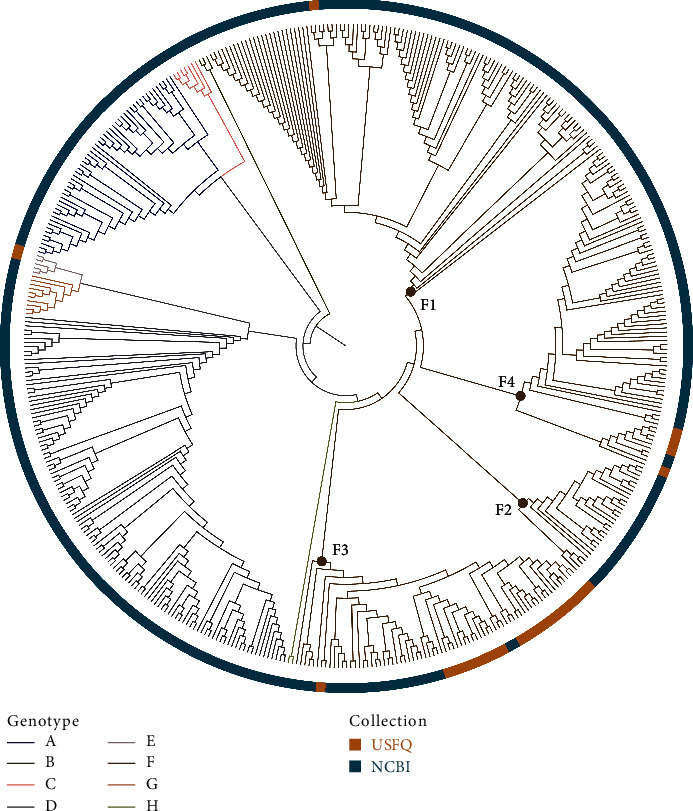
Maximum likelihood phylogenetic tree of South American HBV isolates sourced from NCBI GenBank and Ecuadorian isolates obtained in this study. The tree was constructed using IQ-tree 2.2.0. For all isolates, the preS gene region was extracted from the whole genome. An HBV sequence from genotype J (AB486012) served as the outgroup (not shown). Genotypes are color-coded as follows: A (red), B (brown), C (light green), D (green), E (turquoise), F (blue), G (violet), and H (pink). Collection colors are represented by USFQ (orange) and NCBI (turquoise).

HBV genotype F was predominant in this sample (*n* = 42, 95%), followed by genotype E (*n* = 2, 4%). Of the participants with genotype F, the subgenotypes were primarily F3 (*n* = 35, 83%), followed by F4 (*n* = 6, 14%) and F1b (*n* = 1, 2%) (Figure 1).

**Figure 2:**
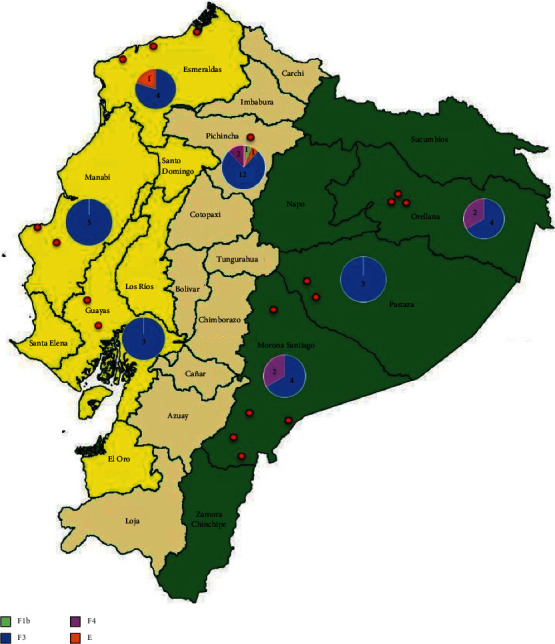
Geographical distribution of genotypes and subgenotypes in Ecuador. Red dots identified the specific sites where subjects were living.

The prevalence of each HBV subgenotype by region is shown in Figure 2. Subgenotype F3 was predominant in the Coastal region (92%), while subgenotypes F3 and F4 were most prevalent in the Amazon region (73% and 26%, respectively). The distribution of HBV genotypes and subgenotypes was more varied in the Andean region, including genotype E (6%) and subgenotypes F1b (6%), F3 (75%), and F4 (13%). Genotype E was also identified in samples from an Afro-Ecuadorian subject from Esmeraldas (Coastal region) and another mestizo from Quito (Andean region).

## 4. Discussion

This study is the first to identify the distribution of HBV genotypes and subgenotypes in an Ecuadorian population. In the study sample, genotype F was most prevalent, followed by genotype E. This finding aligns with that of Velkov et al. [6], who observed that genotype F is predominant in South America, including Colombia [14], Venezuela [15], Peru [16], and Bolivia [17]. Genotype F is also prevalent in Central America [18, 19] and is associated with higher mortality than genotypes A or D [20]. A strong association has been demonstrated between genotype F and the Native American, or Amerindian, populations [21]. These peoples, commonly called “mestizo,” are strongly represented in South America, particularly in Ecuador [22].

The subjects in the current study were phylogenetically similar to cases reported in the neighboring countries of Colombia [14] and Venezuela [15]. Subgenotype F3 formed a monophyletic group in a mixed clade alongside sequences from Ecuador, Chile, Bolivia, Argentina, and Venezuela. A second clade was composed of sequences from Argentina and Bolivia (Figure 1) (supplementary nucleotide alignment HBV). In the phylogenetic analysis, subgenotype F4, identified in the Ecuadorian samples, was closely related to that reported in Bolivia [17] and Argentina [23, 24] (Supplementary Figure 1) (supplementary nucleotide alignment subgenotype F4). In addition, subgenotype F1b from the Ecuadorian samples was phylogenetically similar to sequences reported in Argentina [23, 24], Colombia [25], and Peru [16].

Genotype E was identified in two samples and exhibited a phylogenetic correlation with sequences from West African countries such as Namibia, Gambia, Nigeria, and Cameroon [26], as well as isolates from the Quibdo Department of Colombia [27] (Supplementary Figure 2) (supplementary nucleotide alignment genotype E).

Ecuador, Guyana, Suriname, and French Guiana have remained the only countries in the region for which an HBV genotyping study has not been performed. However, Ecuador was ranked by the Pan American Health Organization [7] as a country with a high HBV prevalence, surpassed only by Peru and Colombia.

A total of 2.1 million people are living with HBV infection in Latin America and the Caribbean. While genotype F is highly prevalent in this region, HBV drug treatment effectiveness assays have primarily focused on genotypes most common in Europe and the United States. Thus, information on the drug susceptibility of genotype F remains limited. Patients with HBV genotype F infection are shown to respond well to interferon treatment [28].

## 5. Conclusions

The current study had some limitations, including its small sample size. However, it is the first study to conduct a nationwide analysis of HBV genotypes distributed in Ecuador using a standardized methodology. The findings identify the most common HBV genotypes and subgenotypes found in the country. Genotype F and subgenotype F3 were the most prevalent, and subgenotype F4 was most likely associated with transmission in Amazon communities. This study highlights the importance of active epidemiological and laboratory surveillance in Ecuador. However, larger studies are needed to further characterize the HBV epidemiology and transmission dynamics in Ecuador and inform the development of targeted prevention and control strategies.

## Figures and Tables

**Table 1:** Sociodemographic characteristics of HBV Ecuadorian patients.

Age	Number	Percentage
18–29	16	36.4
30–65	23	52.3
>65	5	11.3

*Ethnic group*		
Afro-American	8	18.2
Indigenous	7	15.9
Montubio	1	2.3
Mestizo	28	63.6

*City of residency/province of residency*		
*Andean region*		
Quito/Pichincha	16	36.4

*Coast region*		
San Lorenzo/Esmeraldas	3	6.8
Rio Verde/Esmeraldas	1	2.3
Esmeraldas/Esmeraldas	1	2.3
Portoviejo/Manabi	5	11.3
Guayaquil/Guayas	3	6.8

*Amazon region*		
El Coca/Orellana	5	11.3
Loreto/Orellana	1	2.3
Puyo/Pastaza	3	6.8
Yukitais/Morona Santiago	1	2.3
Patuca/Morona Santiago	1	2.3
Logroño/Morona Santiago	2	4.5
Tiwintza/Morona Santiago	1	2.3
Gualaquiza/Morona Santiago	1	2.3

Describing age by age group, ethnic group, and city of residency/province of residency.

## Data Availability

All data and materials are fully available as supplementary material, and sequencing information can be accessed by NCBI accession numbers included in the methods section.

## Supplementary Materials

Supplementary Materials Supplementary table 1: Sample code and corresponding accession number at NCBI. Supplementary nucleotide alignment HBV (Phylogenic tree HBV Ecuador). Supplementary figure 1 (Phylogenic tree F4 subgenotype). Supplementary nucleotide alignment subgenotype F4. Supplementary figure 2 (Phylogenic tree E subgenotype). Supplementary nucleotide alignment genotype E.
