# Supplementary figures and images for: Hepatitis B Virus (HBV) Genotypes in an Ecuadorian Population: A Preliminary Study
Source: Adv Virol. 2024 Aug 23;2024:8823341. doi: 10.1155/2024/8823341 (PMC11364474; doi:10.1155/2024/8823341)

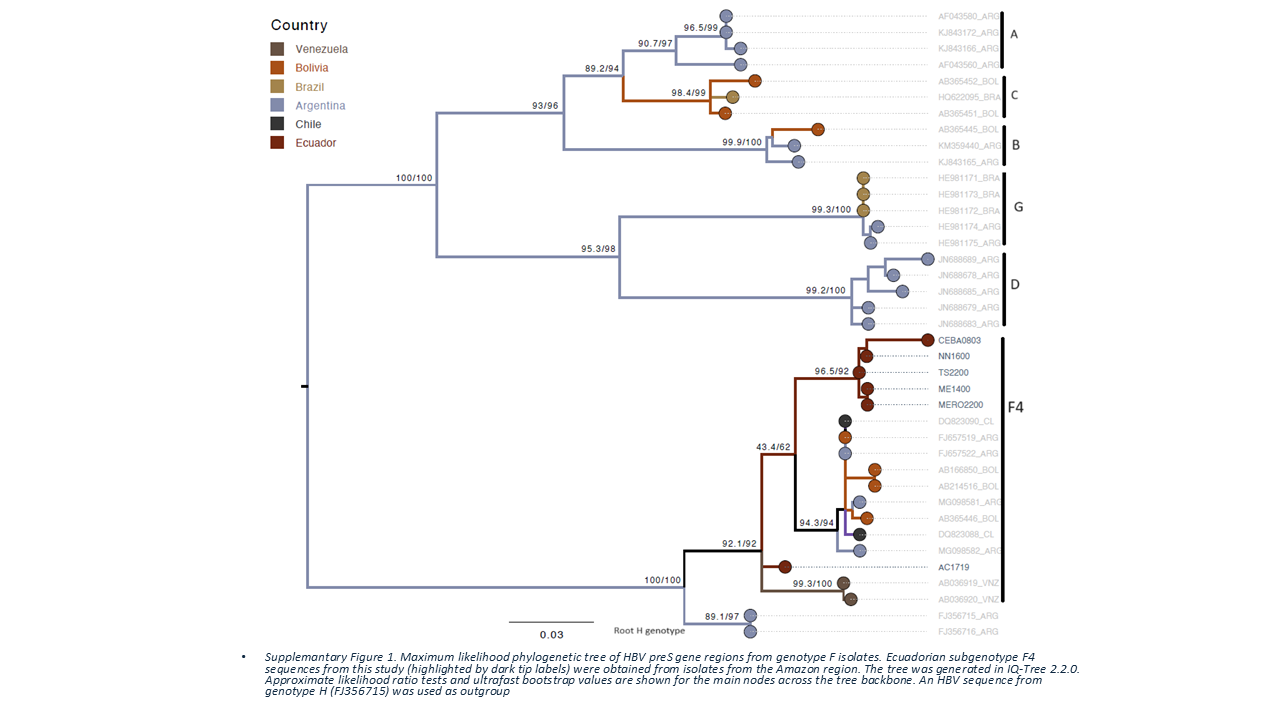

Supplement: Supplementary Materials — Supplementary table 1: Sample code and corresponding accession number at NCBI. Supplementary nucleotide alignment HBV (Phylogenic tree HBV Ecuador). Supplementary figure 1 (Phylogenic tree F4 subgenotype). Supplementary nucleotide alignment subgenotype F4. Supplementary figure 2 (Phylogenic tree E subgenotype). Supplementary nucleotide alignment genotype E. [file 8823341.f1.zip › Supplemantary figure 1.png]

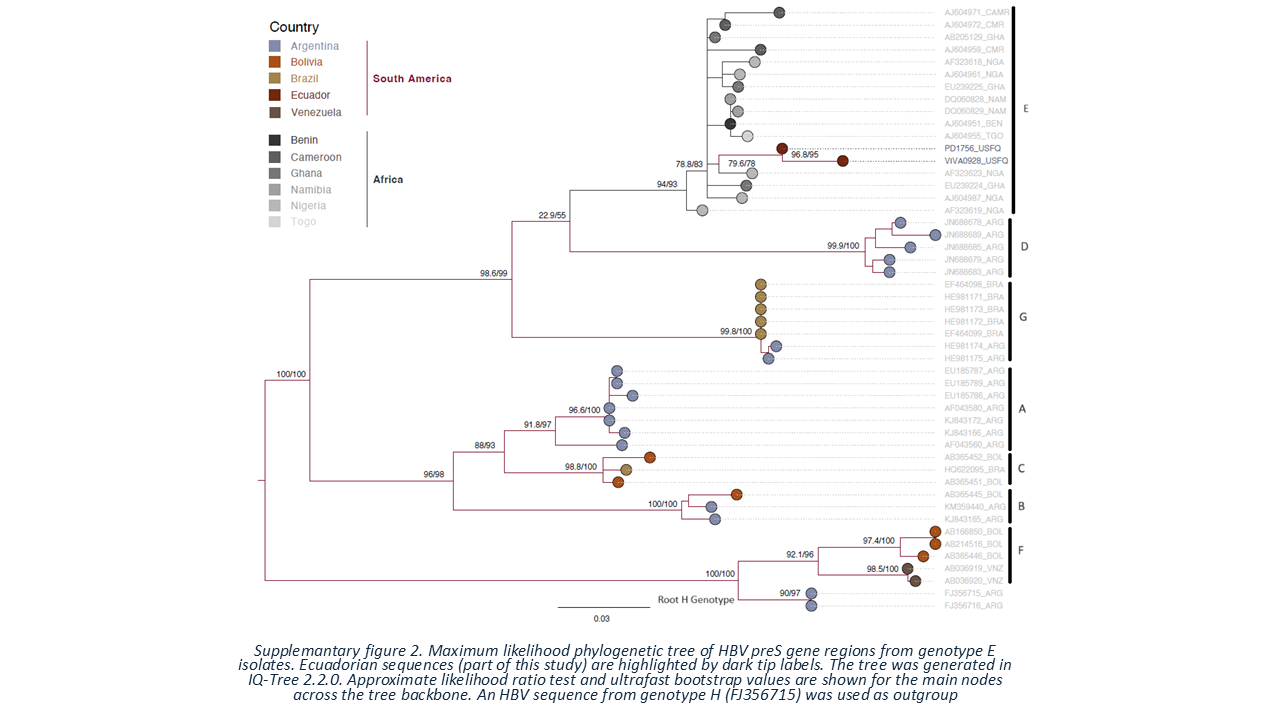

Supplement: Supplementary Materials — Supplementary table 1: Sample code and corresponding accession number at NCBI. Supplementary nucleotide alignment HBV (Phylogenic tree HBV Ecuador). Supplementary figure 1 (Phylogenic tree F4 subgenotype). Supplementary nucleotide alignment subgenotype F4. Supplementary figure 2 (Phylogenic tree E subgenotype). Supplementary nucleotide alignment genotype E. [file 8823341.f1.zip › supplemantary figure 2.png]
